# Institutional Factors Associated with Infection Prevention and Control Practices Globally during the Infectious Pandemics in Resource-Limited Settings

**DOI:** 10.3390/vaccines10111811

**Published:** 2022-10-27

**Authors:** Adil Abalkhail, Thamer Alslamah

**Affiliations:** Department of Public Health, College of Public Health and Health Informatics, Qassim University, Al Bukayriyah 52741, Saudi Arabia

**Keywords:** infection prevention and control, healthcare-associated infections, hygiene, limited resources, healthcare workers, public health

## Abstract

Healthcare-associated infections lead to considerable morbidity, a prolonged hospital stay, antibiotic resistance, long-term disability, mortality and increased healthcare costs. Based on the literature, some individual and socio-demographic factors including knowledge, age and length of service or work experience, gender and type of profession influence compliance with infection prevention and control procedures. In addition, organizational culture, which refers to the assumptions, values, and norms shared among colleagues, can influence an individual’s thinking and healthcare workers’ behavior, either positively or negatively. Infection control practices based on the perspective of patients, hospital management and healthcare workers may help develop a better understanding of the factors influencing compliance with infection prevention and control policies and guidelines.

## 1. Introduction

Among the major problems in healthcare systems and organizations are healthcare-associated infections (HAIs), which are a major threat to patients’ and healthcare workers’ safety [1,2]. HAIs can have a negative impact on a person’s quality of life, possibly shorten their life span, and ultimately cost them a lot of money [2,3]. According to the World Health Organization (WHO), there has been an overall increase in the incidences of HAIs around the world in both developed and developing countries, with an annual average prevalence of about 5–10% in patients. There are now approximately 1.4 million people worldwide suffering from HAIs annually [4]. It has been reported that developing nations are 25% more at risk of acquiring HAIs compared to developed countries [5]. One example, a study conducted by Nofal et al. [6], shows that healthcare workers (HCWs) may demonstrate a low level of knowledge of infection prevention and Control (IPC) measures [6].

Among the factors that may affect an HCW’s compliance with the IPC measures in healthcare settings are knowledge, education and training, experience, lack of supplies (alcohol hand rub, a nearby sink, soap, or paper towels), working in an intensive care unit or surgical ward, working at a public, secondary, or tertiary hospital, and caring for a patient exposed to blood or body fluid [2]. Compliance with IPC can be hindered by a high workload, insufficient time, and an inadequate patient-to-nurse ratio. Implementing a diversified strategy for IPC improvement intervention tactics has been proven to lower HAIs and increase HCW compliance [2].

IPC interventions must recognize and address the interaction between host, pathogen, healthcare personnel, and healthcare institutions in order to effectively treat HAIs [7]. Proper healthcare institutions’ work takes place in a challenging environment where they must meet social demands, adhere to cultural norms, goals, and values, as well as adjust to policy and politics in the face of ongoing economic and financial uncertainties [8]. Consequently, changeable institutional climates and organizational cultures are likely to have an impact on an organization’s ability to execute improvement initiatives, adapt, and survive [9]. 

A serious public health concern is antimicrobial resistance (AMR). AMR refers to the immunity of microbes to a pharmaceutical agent that kills or prevents the growth of microbes, as for example, antibiotic, antibacterial, antiviral, antifungal and antiparasitic. AMR is a threat to the efficiency of antimicrobials in the treatment of infections. Therefore, the excessive consumption, increased frequency and careless use of antimicrobials are likely to result in antimicrobial resistance [10,11,12]. The concern that the WHO raised is that antibiotic resistance can prevent effective infection control [13]. The prevalence of antibiotic-resistant bacteria, the complication of therapy, and the aging of the population are among variables that raise the probability of HAIs [14]. 

Many factors have been associated with non-compliance with infection control procedures globally, including uncertainty among HCWs about infection prevention-related issues such as monitoring and reporting standards, readiness and competence to implement policies and countering outbreaks [15].

## 2. IPC Policies, Knowledge and Education

IPC policies and guidelines have been found to enhance professional nurses’ experiences and understanding of infection control practices [16]. Effective IPC requires an understanding of HCWs [17]. IPC compliance is hindered by a lack of knowledge of the recommended practices, as well as by a lack of awareness of preventative reasons throughout routine patient care and the potential dangers of transmitting germs to patients [18,19]. Poor compliance is caused by ignorance about the suitability, effectiveness, and application of IPC procedures [20,21]. The keystones of enhancing IPC practices are training and education in order to overcome these obstacles [22]. HCWs need to understand the power of knowledge. Hence, knowledge and education of IPC measures play an important role in preventing infection among HCWs [22,23].

The knowledge of HCWs should cover topics such as hand washing, wearing private defensive equipment, vaccination for the preventative measures of infectious diseases, methods of spreading infection, spotting infections among patients, decontaminating medical devices, ability to handle hazardous material, and needle stick and sharp safety regulations. Even more crucially, HCWs must adhere to these IPC precautions, techniques, and approaches to guarantee the decrease in HAIs in hospital settings [20]. Edet et al. 2017 conducted a qualitative study which aimed to understand and explain behaviours that occur in everyday infection control practice from the perspective of healthcare workers. The study used vignettes developed from nurses’ account of practice in order to assess behaviours related to IPC. It has been discovered that workplace policies and infection monitoring improve professionals’ perceptions of IPC procedures as they concentrate on organisational aspects to create a favourable compliance orientation and create long-lasting IPC procedure methods [2].

Participants did not always follow IPC policies and procedures, despite having a clear aim to present themselves as experienced practitioners. This research reveals reasons for non-adherence to protocols, even when practitioners are aware of them, which is an important discovery [24]. For example, some nurses made judgements about their own behaviours which allowed not following guidelines while describing IPC behaviour in terms of “a show” to appear in a good light. The study highlights the need for multifaceted interventions, which the current policy and guidance do not completely provide. The study’s findings suggest that a number of variables affect behavior related to infection control and that nurses’ patchy adherence to recommended procedures is not only attributable to a lack of infection control training. As such, educational interventions should not only consider scientific knowledge but also beliefs, values, and social understandings of infection. Evidence on the role of knowledge, education and attitudes with respect to infection control is contradictory. Some researchers have argued that level of education determines level of knowledge, and that a higher level of knowledge positively influences attitudes and orientation towards compliance with hospital IPC practices. 

Therefore, compliance amongst more highly educated professionals would be expected to be higher than amongst new graduates. However, these assumptions are mostly based on qualitative studies conducted in the United States [25,26], and studies conducted in other contexts and settings may contribute to existing knowledge by showing the extent to which these findings can be more broadly generalized. For instance, a study conducted in the Kingdom of Saudi Arabia (KSA) found that a significant percentage of healthcare practitioners do not adhere to Ventilator-associated pneumonia (VAP) prevention guidelines, with factors related to the organisation, management protocol, and healthcare providers being identified as the main cause of lack of adherence, rather than a lack of education [27]. However, a study by [28] assessed the barriers that affect nurses’ adherence to IPC guidelines, finding that hospital education and the nursing curriculum were the main barriers to adherence to VAP guidelines [29]. Finally, a study by Bassuni and Bayoumi [30] found a need for continuous education programs for intensive care units’ (ICUs) nursing staff in KSA to increase knowledge, and its implementation, of the importance of improving patient safety measures in a complex care context [31]. 

However, factors other than level of education and knowledge can also have an impact on adherence to IPC measures. A study by Jain et al. 2012 found that despite the majority (76%) of participants being aware of IPC measures, only 56% complied with the requirement to dispose of used needles and syringes in puncture-resistant containers. In addition, only 50% of participants observed the requirement not to recap needles, despite only 19% of participants having been immunized against Hepatitis B [32,33]. Given that these findings are based on self-report accounts; it is possible that actual adherence rates may be much lower. Another limitation of this study is that no attempt was made to determine the way in which practitioners rationalized their lack of commitment to policies and procedures. Poor IPC governance at national and facility levels is a common problem for hospitals; a lack of political will results in a dearth of national IPC policies, inadequate funding for IPC activities and dedicated employees, and a lack of resources [34,35]. Future studies in this area could provide valuable insights to inform policymaking.

## 3. Inadequate Infrastructure and Personnel

The availability of suitable facilities for IPC and adequate supply chains for goods such as hand sanitizers and protective gear like gloves and vaccines is another issue that may affect adherence to IPC practices. The execution of high-risk procedures in settings with insufficiently qualified staff can exacerbate a lack of infrastructure for IPC [27]. In second world countries, adequate funds tend to be directed to health institutions within urban areas which have more speciality hospitals. Rural healthcare institutions tend to lack the supplies required to mitigate infections associated with sterile body sites and mucous membranes [33]. It is commonly known that it is difficult to create successful IPC programs in environments with limited resources. Furthermore, a lack of infrastructure, especially insufficient Hygiene facilities, plagues many hospitals [34,36]. Deficiency of IPC training for staff members and lax adherence to IPC measures, such as hand cleanliness, might make staff shortage problems worse [35,36]. Insufficient infection-monitoring systems and overpopulation have also been identified as major barriers to successful IPC in settings with limited resources [36]. Most hospitals have inadequate supplies of gloves, facemasks, disposable and non-disposable aprons, culture bottles, soap dispensers, waste disposal plastics and bins, hand sanitisers and other supplies vital for compliance with IPC practices [37]. Most hospital wards have few single rooms, and most are not equipped with specific isolation rooms. Wards are often inadequate for purpose, with medical wards, including ICUs, being used for surgical purposes. Wards often lack temperature and humidity control equipment and have little in the way of diagnostic microbiology services. In many cases, wards are cramped with insufficient bed-to-bed space and a very high turnaround time for patients, leading to a prevalence of pathogens, such as viral and bacterial infections. Bed-to-bed distance is at least 150 cm in ICUs in the Netherlands, whereas it is generally only 100 cm in most hospitals in Turkey [38]. Inadequately trained staff in ICUs further jeopardizes hand hygiene because compliance requires ongoing in-service training. Increased patient and staff movement inside and between wards raises the likelihood that multidrug resistant (MDR) germs will spread [39]. 

## 4. Ineffective Antibiotic Policies

In order to prevent and control infections, particularly HAIs, effective environmental cleanliness must be maintained [40,41]. Hospital facilities that are contaminated are a major source of microorganism transmission. Hence, keeping good sanitary standards within hospitals is crucial to lowering HAIs [41]. The goal of such hygienic practices is to reduce the number of microbial pathogens that are frequently present on surfaces, because the reduction of pathogens lowers the likelihood that highly contagious pathogens will be transferred from an object to a service user, thereby lowering the risk of cross-infection [42,43]. Mucus, urine, blood, secretions, by-products, bacteria, and dust that might encourage the development of microbes are just a few of the contagious and infectious materials that must be physically removed from surfaces in hospitals using detergents, chemical disinfectants, and water [44]. Despite the numerous studies demonstrating the effectiveness of education programs in supporting changes in antimicrobial prescribing that lead to a decrease in use, such programs are rarely implemented in underdeveloped nations [45].

## 5. Poor Waste Management

The safety of healthcare providers, patients, and society is seriously threatened by improper waste management throughout the industry. Proper health care waste management (HCWM) is frequently difficult in underdeveloped nations [46]. Adverse health and environmental repercussions might result from improper management of waste in the healthcare industry [47]. Communicable diseases transmission has the possibility of occurring at every stage of the HCWM system (separation, storing, transportation, therapy, and waste) [47,48]. Healthcare professionals, patients, and even communities can all suffer from poor HCWM, particularly in low- and middle-income nations [46]. Additionally, improper medical waste management, such as burning waste in the open, creates significant environmental dangers by emitting toxic gases into the neighborhood [49,50].

Wastes from the healthcare industry can be classified as hazardous or non-hazardous; toxic substances, sharp objects, biohazardous material, medicinal wastes, and radioactive trash are examples of hazardous materials [48]. Dressings, needles, wasted sample of tissue, blood microscopy slides, and single-use medical gadgets are all examples of waste that contains blood or other bodily fluids, cultures from laboratory testing, and waste products from patients [17]. Healthcare facilities must have strict policies regarding the handling and disposal of dangerous hospital waste, including infectious waste, as well as cutting objects and other sharp items, such as needles. A study conducted by the WHO, 2018 in twenty-two developing countries reported that the percentage of facilities not using proper waste disposal methods ranged from 18–64%. Recent studies have also shown that the quantity of healthcare waste has risen sharply in recent years, accompanied by inadequate approaches to waste management caused by lack of training, lack of adequate resources, staff resistance, lack of awareness, negligence, poor leadership and unfavorable attitude [51]. Furthermore, a recent report from the United States CDC indicated that there are approximately 385,000 needle stick and sharp object-related injuries amongst healthcare workers annually in the US [52,53]. According to the data provided by WHO, there are approximately 36 million healthcare workers worldwide, of whom around 3 million per year receive an injury with a sharp instrument, resulting in 2,000,000 subjects contaminated with HBV and 1,000,000 with HCV [54]. Furthermore, the WHO reported in 2000 that improper handling, disposal, and reuse/recycling of contaminated syringes and other waste products caused 260,000 HIV infections worldwide (5% of all new infections), 2 million Hepatitis C infections, and 21 million Hepatitis B infections, accounting for 40% of all new infections [46]. The healthcare waste collection and handling system, including bins and containers for separate wastes, is typically in disrepair according to a study undertaken in Cameroon [46].

## 6. Poor Medical Staff Practices

Healthcare professionals’ lack of awareness or understanding of the spread of infectious diseases and the senior management’s absence of commitment [55] are considered areas of concern. This is especially true in underdeveloped nations, where nurses, physicians, and patients frequently are not aware of the significance of IPC and its connection to providing adequate treatment [56]. Medical professionals may have a propensity to focus on patient needs and be unwilling to consider them as a group, a perspective that runs counter to the fundamental ideas of IPC [57]. They frequently think that reducing the dangers of nosocomial infection is unnecessary and that doing so would be impossible [58]. On the contrary, nurses are educated to care for patients in groups and have closer interaction with patients. Nurses are more likely to be carriers of cross-transmission, which increases their risk, but they also tend to view IPC measures favorably. Senior medical staff attitudes may exacerbate the issue of insufficient IPC through personality conflicts, reluctance to change or improve, and inability to collaborate with other healthcare professionals [59].

Several poor nursing practices have been associated with the transmission of HAIs. These include not wearing gloves before touching a patient with an infectious disease, not wearing facemasks or aprons, and not disposing of used gloves, disposable aprons and other infectious waste in proper bins. Other examples of poor nursing practices include recapping needles, the reuse of unsterilized needles, infusing unscreened blood or blood products, and inappropriate use of medical devices. According to the International Labour Organization (ILO), nurses are exposed to needle stick injuries more than other healthcare workers, and needle stick injuries are the most frequent occupational injury amongst nurses [60]. Inappropriate and outdated injection practices, such as the reuse of needles and syringes and the use of multi-dose vials have been implicated in the transmission of HAIs [13]. Despite the availability of needle safe systems and disposal sharp containers, needle stick injuries are still common even in resource-rich settings [61].

An examination of the prevalence of death and disability from injection-associated infections caused by Hepatitis B virus (HBV), Hepatitis C virus (HCV) and HIV found that patients received an average of 3.4 injections per year. A total of 39.3% of these injections were found to be administered with reused equipment [62]. Furthermore, the WHO has identified more than 70 countries which do not test all donated blood for HIV, Hepatitis B, Hepatitis C and syphilis [63]. The majority of HAIs are associated with the inappropriate use of medical devices. For instance, pneumonia has been linked with mechanical ventilation, and UTIs are associated with use of urinary catheters. Surgical Site Infections (SSIs) are caused primarily by surgery, and bacteraemia mainly occurs after the use of intravascular devices [43]. Khan et al. found that UTIs constitute 30–40% of all nosocomial infections, and that ≥25% of patients admitted to ICUs in developed countries develop a HAI, with mortality from HAIs running at >25%. In developing countries, 66% of patients admitted to ICUs develop HAIs [43].

## 7. Poor Food Hygiene and Access to Potable Water

Tap water and food can function as potential reservoirs of nosocomial pathogens. Studies have reported that in most developing countries, hospital water supply is contaminated or interrupted. Waterborne diseases are primarily caused by fecal pollution of water sources. However, in hospitals this problem is compounded due to the nature of the patients and the invasive surgical procedures that occur, which leads to the pollution of water with non-faecal Gram-negative bacteria and Legionella [64]. Food pollution can also occur, with many hospitals failing to monitor the quality of food. Lapses in food-handling are common including, for instance, cooking or storing food at the wrong temperature and the cross-contamination of raw and cooked food, resulting in nosocomial food-borne infections. Research has estimated that the incidence of nosocomial diarrhea in developing countries may be higher than generally believed [65].

## 8. Poor WASH (Water, Sanitation, and Hygiene)

WASH (water, sanitation, and hygiene) are essential healthcare initiatives [66,67]. The proper operation of any healthcare facility (HCF) depends on having access to sufficient quantities and quality of water, as well as the availability of facilities for appropriately treating excreta and medical waste [68]. It has long been known that birth attendants’ hand hygiene habits contribute to invasive infections [69]. Nevertheless, relatively new studies have connected inadequate access to water and sanitation, unhygienic birth conditions, and rising antimicrobial resistance to newborn sepsis and maternal death [70,71]. The majority of IPC practices require the use of WASH procedures and services which are also crucial for raising the level of care provided [68].

The importance of WASH in HCFs has gained more prominence in recent times. Included in this is a worldwide demand for action on WASH in HCFs that was released in 2018 [72] and aims to ensure that everyone has access to high-quality WASH in HCFs, especially in low-income and middle-income countries where services are typically absent. WHO and UNICEF are driving the execution of a worldwide plan to enhance WASH facilities in HCFs with assistance from over 35 partners. Eight actionable initiatives have been identified, including making nationwide blueprints and goals, enhancing maintenance and infrastructures, and involving populations [73].

## 9. Limitations

Within this review, the studies reviewed were non-intervention studies found about prevention and control, only examining the views of HCWs. The topic of HAIs has many facets, so it was not possible to cover them all in one paper; thus, we have selected the most important aspects to highlight.

## 10. Conclusions

Studying IPC practices based on the perspective of patients, hospital management and HCWs may help develop a better understanding of the factors influencing compliance with IPC policies and guidelines. While increasing funds would evidently help to solve some of the above-mentioned problems, this is naturally not a solution for countries with limited resources. It is important to note that although numerous studies have described interventions to support patient involvement in the implementation of IPC guidelines [74,75,76], future studies are still needed to determine how to create and sustain an ‘accepting culture,’ where patient involvement is seen as an integral part of patient safety rather than a personal challenge for healthcare providers [74].

## Data Availability

Not applicable.
